# Zwitterionic Chitosan Derivative, a New Biocompatible Pharmaceutical Excipient, Prevents Endotoxin-Mediated Cytokine Release

**DOI:** 10.1371/journal.pone.0030899

**Published:** 2012-01-24

**Authors:** Gaurav Bajaj, William G. Van Alstine, Yoon Yeo

**Affiliations:** 1 Department of Industrial and Physical Pharmacy, Purdue University, West Lafayette, Indiana, United States of America; 2 Department of Comparative Pathobiology, Purdue University, West Lafayette, Indiana, United States of America; 3 Weldon School of Biomedical Engineering, Purdue University, West Lafayette, Indiana, United States of America; University of Helsinki, Finland

## Abstract

Chitosan is a cationic polymer of natural origin and has been widely explored as a pharmaceutical excipient for a broad range of biomedical applications. While generally considered safe and biocompatible, chitosan has the ability to induce inflammatory reactions, which varies with the physical and chemical properties. We hypothesized that the previously reported zwitterionic chitosan (ZWC) derivative had relatively low pro-inflammatory potential because of the aqueous solubility and reduced amine content. To test this, we compared various chitosans with different aqueous solubilities or primary amine contents with respect to the intraperitoneal (IP) biocompatibility and the propensity to induce pro-inflammatory cytokine production from macrophages. ZWC was relatively well tolerated in ICR mice after IP administration and had no pro-inflammatory effect on naïve macrophages. Comparison with other chitosans indicates that these properties are mainly due to the aqueous solubility at neutral pH and relatively low molecular weight of ZWC. Interestingly, ZWC had a unique ability to suppress cytokine/chemokine production in macrophages challenged with lipopolysaccharide (LPS). This effect is likely due to the strong affinity of ZWC to LPS, which inactivates the pro-inflammatory function of LPS, and appears to be related to the reduced amine content. Our finding warrants further investigation of ZWC as a functional biomaterial.

## Introduction

Chitosan is a linear copolymer of D-glucosamine (2-amino-2-deoxy-D-glucose) and N-acetyl-D-glucosamine (2-acetamido-2-deoxy-D-glucose), obtained by partial (usually>80%) deacetylation of chitin, the main component of exoskeletons of insects and crustaceans [1]. Chitosan has low oral toxicity (oral LD_50_: >10,000 mg/kg in mouse and >1500 mg/kg in rats [2], [3]) and has been used in dietary supplements. It is also known to be safe for topical use and as an ingredient of medical devices or cosmetics [2], [4], [5]. Therefore, chitosan is considered a safe and biocompatible material, and has been widely explored as a pharmaceutical excipient for a variety of applications such as wound healing [6], [7], surgical adhesives [8], [9], mucoadhesive oral drug/gene delivery [10], [11], gene delivery [12], [13], and tissue engineering [2], [14]–[16].

With a pKa of ∼6.5, chitosan is insoluble in water at neutral pH, where the majority of amines are deprotonated, but it is positively charged and water-soluble at acidic pH [11]. The limited solubility of chitosan in neutral pH provides a unique opportunity to form nanoparticulate drug/gene delivery platforms [11], but it is also an obstacle if one intends to apply chitosan as a solution in the physiological condition [13]. To improve chitosan solubility in a broader range of pH, the amine groups of chitosan are partially quaternized [13] or conjugated with a sugar moiety [17]. Glycol chitosan, a chitosan derivative with 2-hydroxyethylether groups in the 6-O position, is also used when aqueous solubility of chitosan at neutral pH is desired [18]–[21]. We have recently reported that chitosan partially amidated with succinic anhydride has a unique pH-dependent charge profile, with isoelectric points (pI) tunable from pH 5 to 7 [22]. The chitosan derivative, which we call zwitterionic chitosan (ZWC), is soluble in water at pH's below and above the pI according to the change of its net charge.

Due to the unique pH dependence and aqueous solubility, we propose to use ZWC for parenteral applications, specifically as a component of nanoparticulate drug delivery systems. For nanoparticles or the components to be compatible with parenteral applications, they should not activate immune cells in the bloodstream (monocytes, platelets, leukocytes, and dendritic cells) and in tissues (resident phagocytes), which would cause premature removal of the nanoparticles and/or elicit inflammatory responses [23]. There are split opinions about biocompatibility of chitosan. Some studies indicate that chitosan has various biological activities that can adversely influence its parenteral applications. Chitosan is shown to cause hemostatic effect and complement activation [2], [24]–[26]. Studies also show that chitosan is an inducer of pro-inflammatory cytokines or chemokines [2], [16], [27]–[32]. Intraperitoneal (IP) administration of chitosan induces a large number of macrophages with hyperplasia in the mesenterium of mice [33] and causes severe peritoneal adhesions in rabbits [27]. On the other hand, others do not observe pro-inflammatory activities of chitosans [34]–[36]. Some studies with chitosan derivatives and chitosan oligosaccharides even find that the chitosans have a protective effect on various cells and prevent cytokine induction [37]–[41]. These studies suggest that the biocompatibility of chitosans may not be generally assumed but varies with the physical and chemical properties of chitosans.

In our previous study, ZWC showed excellent compatibility with blood components and was well tolerated upon IP injection [22]. Compared to its precursor, low molecular weight chitosan (LMCS), the ZWC showed lower potential to cause hemolysis, complement activation, and inflammatory responses [22]. To understand what makes ZWC relatively more compatible with IP application, here we compare ZWC with other chitosans having different aqueous solubilities, primary amine contents, or molecular weights (summarized in **Table S1**), with respect to the tissue reactions to IP-administered chitosans and the propensity to induce pro-inflammatory cytokine production from macrophages. We report that ZWC is not only distinguished from the other chitosans in IP biocompatibility but also possesses a unique ability to suppress pro-inflammatory responses of activated macrophages. These properties are attributable to good aqueous solubility, relatively low molecular weight, and reduced amine content of ZWC.

## Materials and Methods

### Materials

Mouse peritoneal macropahge cell lines were purchased from ATCC (CRL-2457). All media and their components were purchased from Invitrogen (Carlsbad, CA). All other reagents were purchased from Sigma-Aldrich (St. Louis, MO, USA). Chitosan glutamate (Protasan UP G113; MW: 200 kDa; degree of deacetylation: 75–90%) was purchased from Novamatrix (Norway), low-molecular-weight chitosan (LMCS; MW: 15 kDa; degree of deacetylation: 87%) from Polysciences, and glycol chitosan (MW: 82 kDa; degree of deacetylation: 83%) from Wako USA (Richmond, VA).

### Synthesis of ZWC and other chitosans

ZWC was synthesized as previously described [22]. Briefly, LMCS was first dissolved in 1% acetic acid to obtain an acetate salt form. LMCS acetate 200 mg was dissolved in 30 mL of deionized water. Succinic anhydride was added as solid to the LMCS solution under vigorous stirring varying the quantities according to the desired molar feed ratio of anhydride to amine (An/Am ratio). The pH of the reaction mixture was maintained at 6–6.5 and subsequently increased to 8–9 with 1 N NaHCO_3._ After an overnight reaction at room temperature under stirring, the reaction mixture was dialyzed against water (molecular weight cutoff: 3500) maintaining the pH at 8–9 with 1 N NaOH. The purified ZWC was freeze-dried and stored at −20°C. Unless specified otherwise, ZWC prepared with An/Am ratio of 0.7 was used in most experiments.

### Ethics statement

Animals were cared for in compliance with a protocol specifically approved for this study by the Purdue Animal Care and Usage Committee (Approval number: 09-093-11), in conformity with the NIH guidelines for the care and use of laboratory animals.

### *In vivo* biocompatibility

Chitosan glutamate, glycol chitosan, and ZWC were tested for tissue responses following IP administration (800 mg/kg). Chitosan and buffer controls (phosphate buffered saline (PBS), pH 7.4, or glutamate buffer, pH 5) were sterilized by aseptic filtration. Chitosan solutions (20 mg/mL) were prepared by dissolving chitosan glutamate in water or glycol chitosan and ZWC in PBS. ICR mice (25 g) (Harlan, Indianapolis, IN) were anesthetized with subcutaneous injection of ketamine 50 mg/kg and xylazine 10 mg/kg. A 0.5 cm skin incision was made in the skin 0.5 cm above the costal margin, and the peritoneum was nicked with a 24-gauge catheter. One milliliter of 20 mg/mL chitosan solutions or control buffers were injected into the peritoneal cavity through the catheter, and the skin was closed with suture. Animals were sacrificed after 7 days to evaluate the presence of residues, tissue adhesions, and visible signs of inflammation (nodules, increased vascularization) in the peritoneal cavity. Liver and spleen were sampled for histology, and the peritoneal fluid was sampled on a slide for cytological analysis. After fixation in 10% formalin, the sectioned organ samples and peritoneal fluid cells were stained with hematoxylin and eosin (H&E).

### Cell proliferation assay

Mouse peritoneal macrophages were maintained in Dulbecco's Modified Eagle Medium (DMEM) supplemented with 5% fetal bovine serum and 5 mM HEPES. Cells were seeded in 24 well plates at a density of 50,000 cells per well in 1 mL culture medium. After overnight incubation, chitosan solutions (2 or 20 mg/mL) were added to make a final concentration of the medium 0.2 or 2 mg/mL. PBS and lipopolysaccharide (LPS) (1 µg/mL) were added in control groups. MTT assay was performed after 24 hours of incubation to determine the effects of chitosans on macrophage proliferation.

### Cytokine release from peritoneal macrophages

Peritoneal macrophages were seeded in 24-well plates at a density of 150,000 cells per well in 1 mL of medium. After overnight incubation, 100 µL of the chitosan solution was added to each well to bring the final chitosan concentration in medium to 2 mg/mL. In control groups, 100 µL of PBS or glutamate buffer (pH 5) was added in lieu of chitosan solutions. After 24 hour incubation, the culture media were centrifuged at 2000 rpm for 10 min to separate supernatants. The concentrations of interleukin (IL)-1β, IL-6, tumor necrosis factor (TNF)-α, and macrophage inflammatory protein (MIP)-2 in the supernatant were determined using a Milliplex Multi-Analyte Profiling (MAP) cytokine/chemokine panel (Millipore, Billerica, MA). In another set of experiments, macrophages were first challenged by adding LPS to the media in the final concentration of 1 µg/mL shortly before the chitosans or buffer controls. For selected samples, enzyme-linked immunosorbent assay (ELISA) was performed to determine the MIP-2 levels using an MIP-2 ELISA kit (R&D systems, Minneapolis, MN). The detection range of MAP panel was 0–10,000 pg/mL for all analytes. For MIP-2 ELISA, standard curves were prepared in the range of 0–667 pg/mL. In both assays, the supernatant collected from LPS-challenged macrophages was always diluted 10 times prior to the analysis.

To investigate the time course of the ZWC effect on cytokine production, ZWC or LMCS was added in the final concentration of 2 mg/mL at 0, 2, 4, or 8 hours after the LPS addition. After incubating with chitosans for 24 hours, the culture media were collected and diluted 10 times, and the MIP-2 levels were determined using ELISA. For comparison, another set of macrophages was challenged with LPS and incubated for 0, 2, 4, 8, or 24 hours, and the media were sampled without any treatment or further incubation.

To investigate the effect of ZWC on LPS, LPS was preincubated with ZWC before it was added to the cells. Briefly, 10 µg of LPS was mixed with 20 mg of ZWC in 1 mL of 0.9% NaCl and incubated at room temperature for 1 hour. The ratio of LPS to ZWC (10 µg per 20 mg) was consistent with the ratio used in prior experiments (1 µg per 2 mg). ZWC was then precipitated by decreasing the solution pH to 4.8 with 0.1–1 M HCl and removed by 15-min centrifugation at 10,000 rpm. Assuming that the LPS was present in the supernatant, a volume equivalent to 1 µg of LPS was sampled and added to 1 mL of peritoneal macrophage culture. After overnight incubation, MIP-2 levels in the culture media were determined using ELISA.

### Endotoxin analysis

The amount of endotoxin present in each chitosan was determined by the kinetic turbidometric Limulus Amebocyte Lysate (LAL) assay at Associates of Cape Cod Inc. (East Falmouth, MA). Chitosan samples were initially prepared as 1 mg/mL (ZWC, LMCS) or 10 mg/mL (chitosan glutamate, glycol chitosan) solutions in LAL reagent water (LRW) and then serially diluted from 1∶20 to 1∶8000 to find the minimum concentration that did not interfere with analysis. *E. coli* O113:H10 was used as a control standard endotoxin and serially diluted from 0.32 to 0.002 EU/mL to construct a calibration curve. Positive product controls were prepared in parallel by fortifying the diluted samples with additional endotoxin equivalent to 0.008 EU/mL. LRW was tested as a negative control and found to contain less than the lowest concentration of the calibration curve (0.002 EU/mL). Pyrotell®-T LAL lysate was reconstituted with Glucashield buffer, a β-glucan inhibiting buffer, and mixed with samples or controls in a 1∶1 ratio in a depyrogenated microplate. The absorbance of each well was monitored over time. The time required for the absorbance to increase significantly over background was defined as the onset time. The correlation coefficient for the regression of log of onset time vs. log of endotoxin concentration was ≥0.98. All samples were tested in duplicate. The results were reported as the amount of endotoxin present in each chitosan (EU/g).

### Statistical analysis

All data were expressed as mean ± standard deviation. One-way ANOVA was used to determine difference among the groups. Multiple comparisons between treatments were performed with Bonferroni's test, and pair-wise comparison of each treatment with the control group was performed with Dunnett's test A value of p<0.05 was considered statistically significant.

## Results

### Chitosan properties

**Figure 1:**
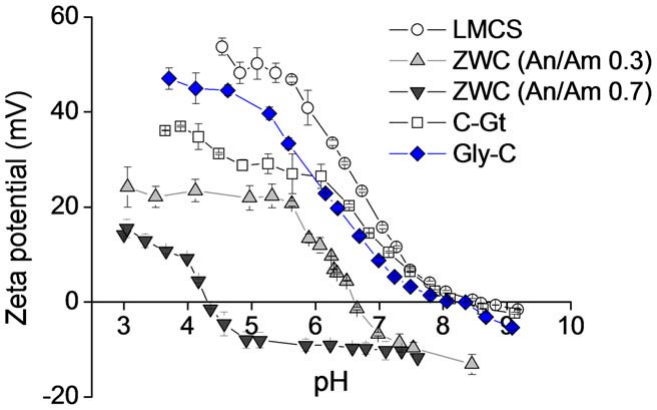
pH-dependent zeta-potential profiles of unmodified low molecular weight chitosan (LMCS) (data from the previous study [**22**]) and zwitterionic chitosan (ZWC) derivatives prepared with different anhydride to amine (An/Am) ratios, chitosan glutamate (C-Gt), and glycol chitosan (Gly-C). Data are expressed as averages with standard deviations of 3 repeated measurements.

All chitosans showed pH-dependence in aqueous solubilities (data not shown) and corresponding charge profiles (**Fig. 1**). Solutions of chitosan glutamate and LMCS (10 mg/mL) became turbid at pH above 6.5 reaching the maximum turbidity at pH 8, where they had neutral charges. On the other hand, ZWC (10 mg/mL) formed clear solutions at both acidic and basic pHs, indicating aqueous solubility, except at the pI value. The pI value of ZWC decreased with the increase of the An/Am ratio (Fig. 1), consistent with our previous report [22]. Glycol chitosan was similar to chitosan glutamate and LMCS in that it showed neutral charges around pH 8, but the solution (10 mg/mL) was not turbid, which indicated the aqueous solubility of glycol chitosan.

### Gross tissue responses to intraperitoneally administered chitosans

**Figure 2:**
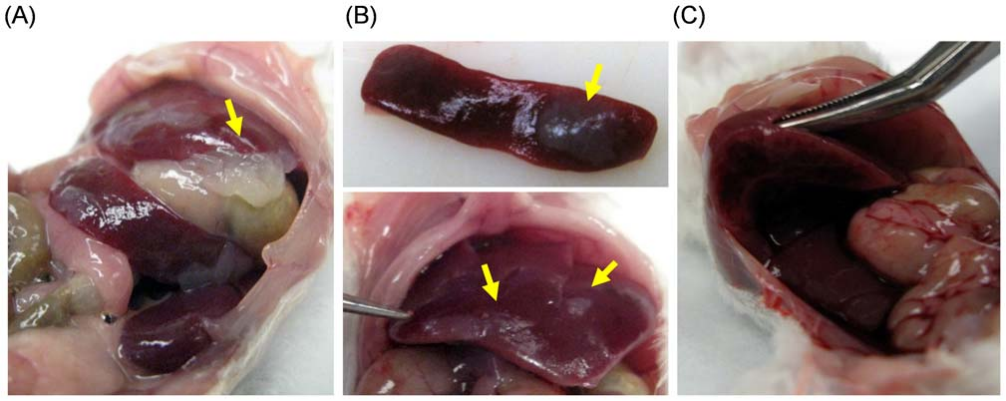
Chitosan precipitates (arrows) in the peritoneal cavity. Mice injected with chitosan glutamate intraperitoneally were examined 7 days after injection. (A) Chitosan precipitates found between the liver and the stomach. (B) Chitosan precipitates stuck on the spleen (top) and the liver (bottom). (C) Lobes of the liver were connected via chitosan residue.

ICR mice received IP injection of chitosan glutamate, glycol chitosan, and ZWC (800 mg/kg). The animals were sacrificed after 7 days, which was found to be an optimal time point for the evaluation of inflammatory tissue responses [42]. During the observation period, the animals did not show any signs of distress and body weight change, similar to those treated with buffer controls. Upon necropsy, the organs of animals treated with ZWC or glycol chitosan were grossly normal. No material was found in the peritoneal cavity of the mouse injected with glycol chitosan or ZWC. On the other hand, white chitosan precipitates were seen in all mice injected with chitosan glutamate due to the near-neutral pH of the peritoneal fluid [43] (**Fig. 2A**). The white precipitates were usually present on the liver and spleen (**Fig. 2B**). In 3 out of 4 cases, lobes of the liver were connected via the residual materials (**Fig. 2C**).

### Histological and cytological evaluation

**Figure 3:**
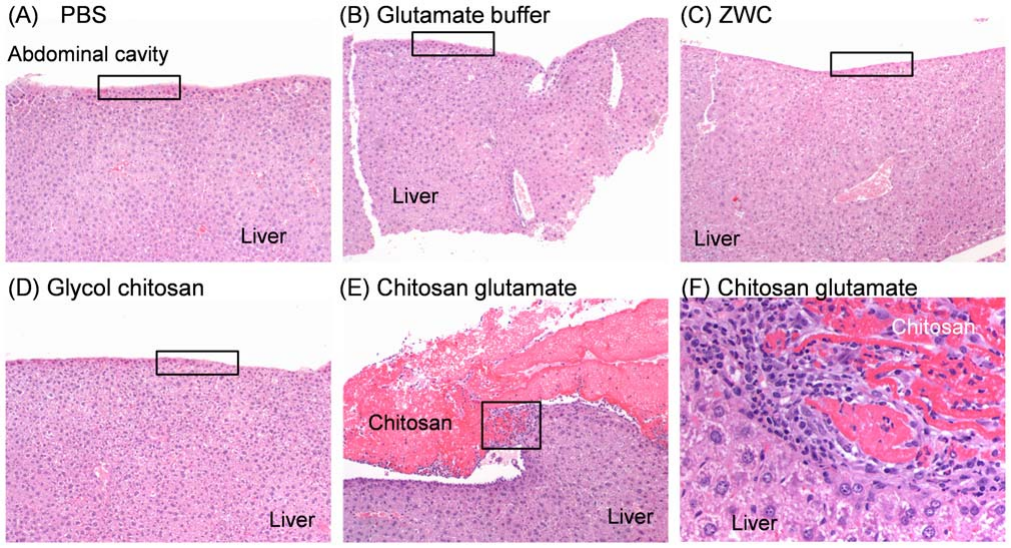
Hematoxylin and eosin staining of liver sections of different treatment groups. (A) PBS (100x); (B) Glutamate buffer (100x); (C) ZWC (100x); (D) Glycol chitosan (100x). (A-D) Normal capsular surface (box). (E) Chitosan glutamate (100x): capsular surface of liver markedly thickened with precipitates of chitosan, which are surrounded by chronic inflammation and mild fibrosis (box). (F) Chitosan glutamate (400x): precipitates of chitosan on the liver surface surrounded by macrophages, fibroblasts, and neutrophils.

**Figure 4:**
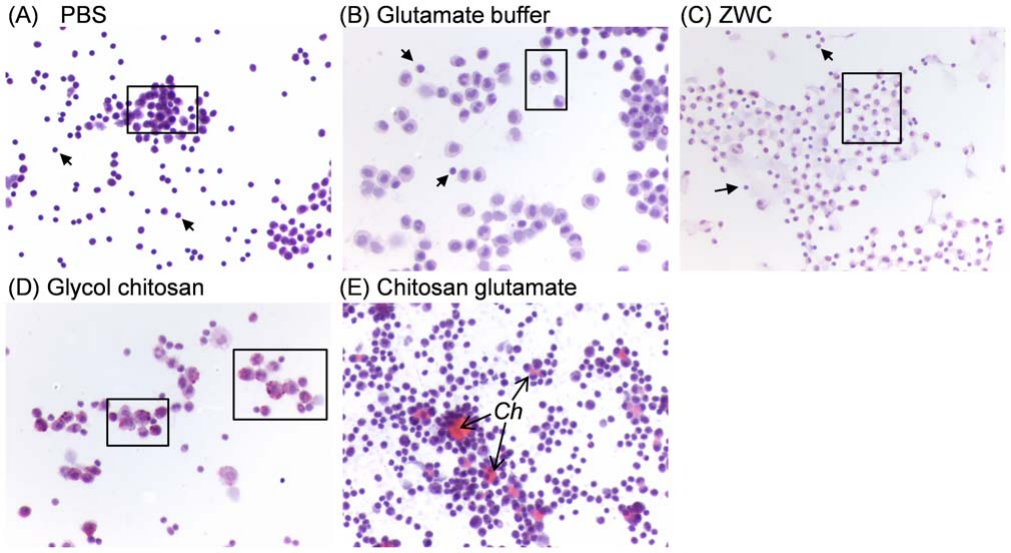
Cytology of the peritoneal fluid from different treatment groups using hematoxylin and eosin staining. (A) PBS; (B) Glutamate buffer; (C) ZWC. (A-C) Peritoneal fluid composed of small macrophages (box) and lymphocytes (arrows). No chitosan precipitates were identified. (D) Glycol chitosan: peritoneal fluid is composed of large macrophages (box) containing chitosan. (E) Chitosan glutamate: peritoneal fluid composed of large macrophages with intracellular eosinophilic chitosan. Extracellular chitosan (Ch) is surrounded by numerous macrophages. All images are of 400× magnification.

Biomaterials delivered to peritoneal cavity often cause inflammatory responses followed by adhesion formation between in peritoneal tissues and abdominal walls [44]. Once entering systemic circulation, they can also cause abnormalities in filtering organs [44]. To estimate the destination and effect of IP chitosan, peritoneal fluid and organs as well as abdominal wall were microscopically examined. Incidence of lesions in peritoneal tissues is summarized in **Table 1**. In mice injected with PBS, glutamate buffer, and ZWC, no significant microscopic differences were seen in the liver (**Fig. 3A, 3B, or 3C**), spleen, and abdominal wall. One mouse treated with glycol chitosan had mild inflammation of the body wall, but liver (**Fig. 3D**) and spleen were normal. Peritoneal tissues from other mice in this group were unremarkable. In contrast, mice treated with chitosan glutamate had noticeable chitosan precipitates on the liver, spleen and abdominal wall, which were surrounded by macrophages and neutrophils **(** **Fig. 3E and 3F**). Capsular surface of the liver adjacent to precipitates of chitosan was thickened and mildly fibrotic (Fig. 3E and 3F). No abnormality was observed in peritoneal fluid of the animals injected with PBS, glutamate buffer, or ZWC (**Fig. 4A, 4B, and 4C**). However, chitosan precipitates were detected in peritoneal macrophages in mice treated with glycol chitosan (**Fig. 4D**) or chitosan glutamate (**Fig. 4E**). Chitosan glutamate was also observed as extracellular residues, surrounded by large activated macrophages (**Fig. 4E**). No chitosan precipitates were observed in those injected with ZWC (**Fig. 4C**).

**Table 1:** Incidence of lesions in tissues after intraperitoneal injection of chitosans and buffers.

	PBS	Glutamate buffer	ZWC (An/Am = 0.7)	Glycol chitosan	Chitosan glutamate
Liver, capsule inflammation	0/2^a^	0/3	0/5	0/4	4/4
Liver, capsular chitosan precipitates	0/2	0/3	0/5	0/4	4/4
Spleen, capsule inflammation	0/2	0/3	0/5	0/4	3/4
Spleen, capsular chitosan precipitates	0/2	0/3	0/5	0/4	3/4
Body wall, inflammation	0/2	0/3	0/5	1/4	3/4
Body wall, chitosan precipitates	0/2	0/3	0/5	0/4	0/4
Peritoneal fluid, inflammation	0/2	0/4	0/5	3/3	4/4
Peritoneal fluid, chitosan precipitates	0/2	0/4	0/5	3/3	4/4

a Incidence of occurrence: Number of mice with lesion/total number of mice examined.

### Chitosan effect on macrophage proliferation

**Figure 5:**
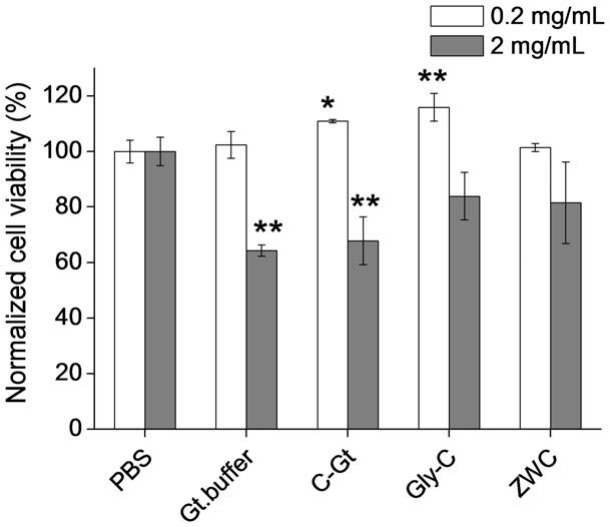
Viability of mouse peritoneal macrophages in the presence of ZWC (An/Am ratio = 0.7), chitosan glutamate (C-Gt) and glycol chitosan (Gly-C). Data are expressed as averages with standard deviations of three repeated measurements. *: p<0.05; **: p<0.01 vs PBS.

In an attempt to understand the difference in IP responses to chitosan glutamate, glycol chitosan, and ZWC, *in vitro* proliferation of peritoneal macrophages was evaluated in the presence of the three chitosans. Peritoneal macrophages were chosen because they are prevalent in the peritoneal cavity and likely to be an important player in inflammatory responses to IP injected chitosans. For all chitosans, 0.2 mg/mL of chitosan treatment did not negatively influence the macrophage proliferation (**Fig. 5**). At 2 mg/mL, there was a moderate reduction in macrophage proliferation with chitosan glutamate (p<0.01 vs. PBS). Glutamate buffer (pH 5) added in an equivalent volume showed a similar level of decrease in cell proliferation, indicating that this reduction might be partly due to the acidity of the medium. Neither glycol chitosan nor ZWC significantly reduced the macrophage proliferation at 2 mg/mL.

### Cytokine induction by chitosans

**Figure 6:**
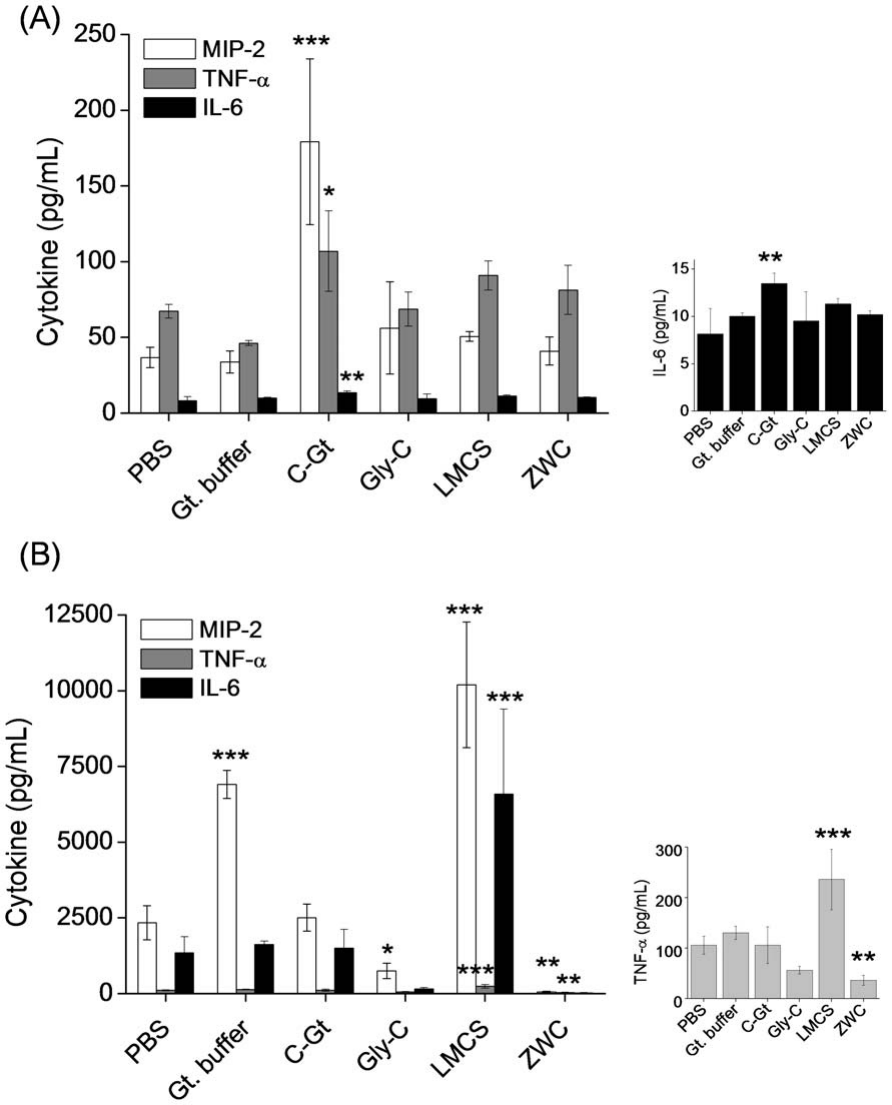
Effect of chitosan treatment (all in 2 mg/mL) on the levels of proinflammatory cytokines released from (A) naïve mouse peritoneal macrophages and (B) LPS-challenged macrophages. Cytokine levels are determined by Milliplex Multi-Analyte Profiling cytokine/chemokine panel. Media of the LPS-challenged macrophages were 10 times diluted prior to analysis. Graphs on the right are displayed in narrow y-scales. ZWC (An/Am = 0.7); C-Gt: chitosan glutamate; Gly-C: glycol chitosan. Data are expressed as averages with standard deviations of three repeated measurements. *: p<0.05; **: p<0.01; ***: p<0.001 vs PBS.

To investigate whether each chitosan had an intrinsic ability to activate peritoneal macrophages, naïve (non-challenged) peritoneal macrophages were incubated with different chitosans (2 mg/mL), and the medium was analyzed to determine the concentrations of pro-inflammatory cytokines (IL-1β, TNF-α, IL-6 and MIP-2). In this experiment, LMCS, the parent material for ZWC, was also tested. Naïve macrophages treated with PBS produced 37±7 pg/mL of MIP-2, 67±5 pg/mL of TNF-α, and 8±3 pg/mL of IL-6, which were considered basal levels of cytokines. There was no additional cytokine release in those treated with glutamate buffer, glycol chitosan, LMCS, and ZWC. There was no difference between LMCS and ZWC-treated groups. On the other hand, chitosan glutamate treatment resulted in significant increases in the levels of MIP-2 (p<0.001), TNF-α (p<0.05), and IL-6 (p<0.01), as compared with PBS-treatment (**Fig. 6A**).

To investigate how each chitosan influenced the cytokine production in activated macrophages, the cells were first challenged with LPS, a potent inducer of cytokine release [45], prior to the addition of chitosans (2 mg/mL). LPS-challenged, then PBS-treated macrophages produced 2341±564 pg/mL of MIP-2, 106±18 pg/mL of TNF-α, and 1346±535 pg/mL of IL-6 (**Fig. 6B**). Glutamate buffer caused increase in MIP-2 production, whereas chitosan glutamate did not have any influence. LMCS treatment increased production of all three cytokines from the LPS-challenged macrophages. Interestingly, ZWC caused a marked decrease in the LPS-induced production of MIP-2 (p<0.01) and TNF-α (p<0.01) as compared with PBS. Glycol chitosan also decreased the production of MIP-2 as compared to PBS. Chitosan treatment did not cause any change in IL-1β levels in either naïve or LPS-challenged macrophages (data not shown).

### MIP-2 induction by chitosans with different number of amine groups

**Figure 7:**
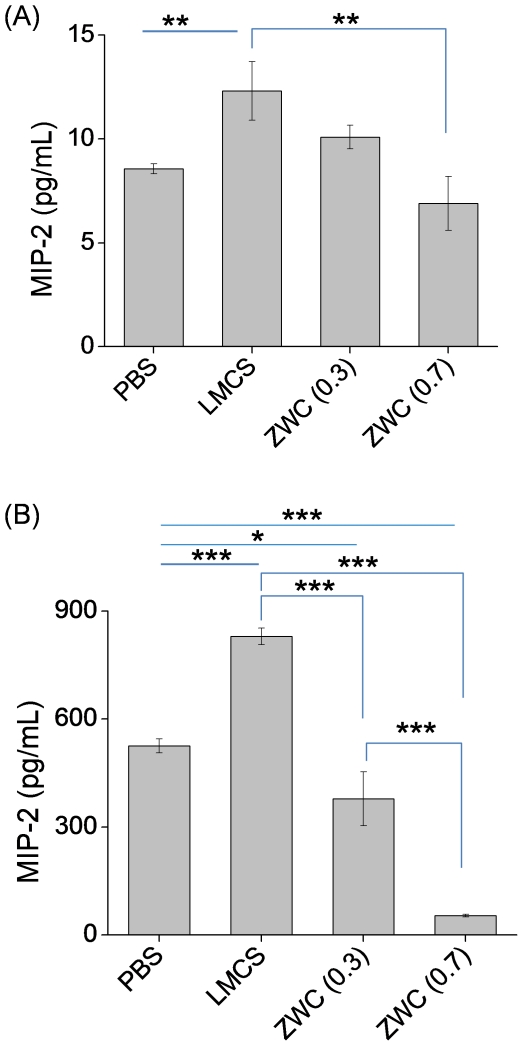
Effect of chitosan treatment (all in 2 mg/mL) on the MIP-2 production from (A) naïve mouse peritoneal macrophages and (B) LPS-challenged macrophages. MIP-2 level is determined by ELISA. Media of the LPS-challenged macrophages were 10 times diluted prior to analysis. Data are expressed as averages with standard deviations of three repeated measurements. **: p<0.01; ***: p<0.001.

The effects of chitosans on MIP-2 release from naïve or LPS-challenged macrophages were monitored varying the amine content in the chitosan. We compared LMCS and ZWC with different An/Am ratios (0.3 or 0.7), all at 2 mg/mL, with respect to the ability to induce macrophages to produce MIP-2, the most sensitive response in the prior experiment. From naïve macrophages, LMCS induced a higher level of MIP-2 than PBS (p<0.01), but no significant change was observed after ZWC treatment (**Fig. 7A**). In LPS-challenged macrophages, LMCS significantly increased the MIP-2 level (p<0.001). In contrast, the two ZWC's suppressed MIP-2 production from the LPS-challenged macrophages (p<0.001 for An/Am: 0.7, p<0.05 for An/Am 0.3 vs PBS) (**Fig. 7B**). ZWC (An/Am: 0.7) decreased the LPS-induced MIP-2 production to a greater extent than ZWC (An/Am: 0.3).

Of note, MIP-2 levels measured by ELISA were not identical to the values determined with the MAP panel, most likely due to the difference between the two assay methods in the sensitive detection ranges. However, results of the two assays were consistent in that MIP-2 levels from LPS-challenged macrophages were at least two orders of magnitude higher than those of naïve macrophages and that the MIP-2 production from the LPS-challenged macrophages was significantly reduced by the ZWC treatment.

### Onset of ZWC effect on LPS-induced MIP-2 production

**Figure 8:**
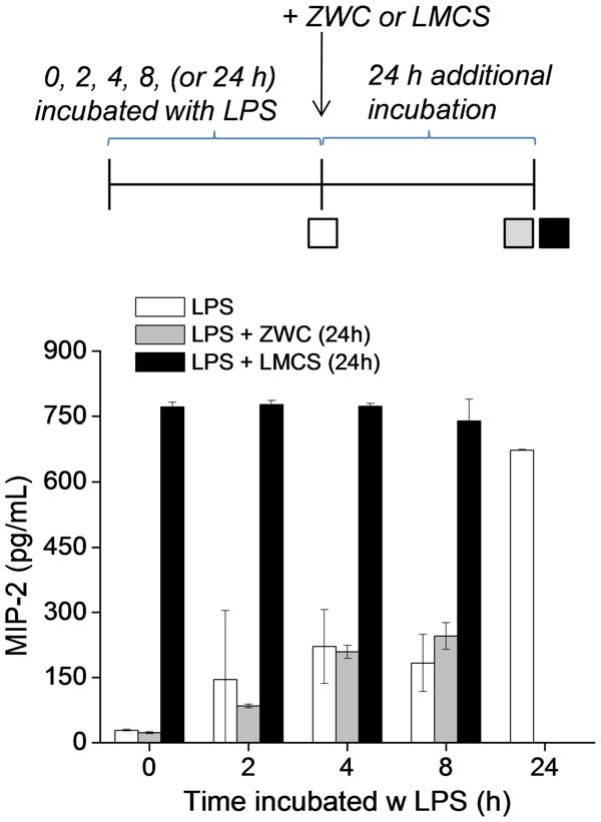
Effect of timed application of ZWC or LMCS (all in 2 mg/mL) on MIP-2 production in the LPS-challenged macrophages. Mouse peritoneal macrophages were incubated with LPS for 0, 2, 4, 8, or 24 hours, and the culture medium was sampled for determination of the MIP-2 level (white bars). In another set, macrophages were incubated with LPS for 0, 2, 4, or 8 hours with LPS and then treated with ZWC or LMCS, and the media were sampled after 24 hours (grey or black bars). Data are expressed as averages with standard deviations of three repeated measurements.

To confirm the ability of ZWC to prevent LPS-induced cytokine production and examine the onset of the action, macrophages were first challenged with LPS for 0, 2, 4, or 8 hours. Subsequently, ZWC or LMCS were added to the challenged macrophages, followed by additional 24 hour incubation. In ZWC-treated macrophages, the MIP-2 levels in the culture media were comparable to those sampled prior to ZWC treatment (**Fig. 8**). This result shows that cytokine production was completely blocked from the time ZWC was added to the medium, and proliferating cells did not further produce cytokines. In contrast, LMCS-treated macrophages continued to produce MIP-2, resulting in the same level as those grown for 24 hours without any other treatment after LPS challenge.

### LPS inactivation by ZWC

**Figure 9:**
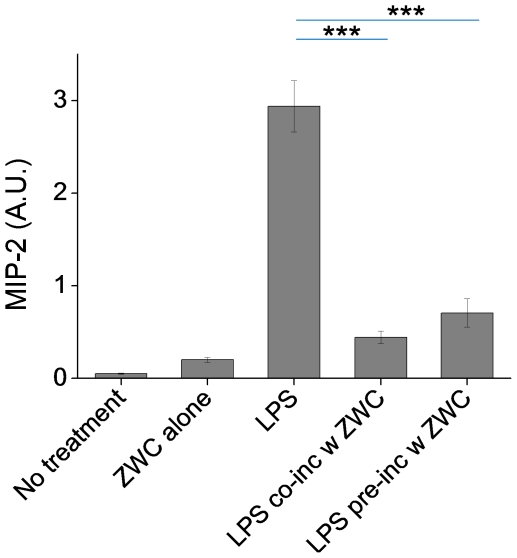
MIP-2 production from macrophages incubated with ZWC (2 mg/mL), LPS (1 µg/mL), a mixture of LPS (1 µg/mL) and ZWC (2 mg/mL) (LPS co-inc w ZWC), or LPS pre-incubated with ZWC (equivalent to 1 µg/mL LPS under an assumption that all the LPS remained in the supernatant; LPS pre-inc w ZWC). ELISA was performed on 10 times-diluted culture media. Data are expressed as averages with standard deviations of three repeated measurements. ***: p<0.001.

To investigate how ZWC prevented the MIP-2 production from the LPS-challenged macrophages, LPS was incubated with ZWC for 1 hour before it was given to the macrophages. ZWC was removed by precipitation at pH 4.8 (∼pI of ZWC) at the end of the 1-h incubation so that the direct effect of ZWC on the cells could be excluded. **Fig. 9** shows that the LPS-induced MIP-2 production was reduced when ZWC coexisted in the culture, consistent with prior experiments. The LPS pre-treated with ZWC also lowered the MIP-2 production to a comparable level. This result suggests that the reduction in MIP-2 production was due to the inactivation of LPS by ZWC rather than a direct effect of ZWC on the LPS-challenged cells. A similar trend was observed with LPS pre-incubated at a higher ratio of LPS to ZWC (30 µg LPS per 20 mg ZWC). Further increase of LPS (40 µg LPS per 20 mg ZWC) resulted in a significant production of MIP-2 (data not shown), indicating that there was an upper limit of LPS that a fixed amount of ZWC could inactivate.

### Endotoxin content in chitosans

We hypothesized that ZWC inactivated LPS by high-affinity interaction. To test this possibility, we determined the endotoxin levels in all chitosans used in this study (**Table 2**). The levels were comparable among chitosan glutamate, glycol chitosan, and LMCS. However, endotoxin levels in ZWC were one or two orders of magnitude higher than those of other chitosans. ZWC with An/Am ratio 0.7 had highest endotoxin concentration. This result suggests the relatively high affinity of ZWC to LPS.

**Table 2:** Endotoxin levels in chitosans.

Sample	Endotoxin concentration (EU/g)
Chitosan glutamate	247
Glycol chitosan	311
LMCS	311
ZWC (An/Am = 0.3)	6,860
ZWC (An/Am = 0.7)	14,150

## Discussion

We previously reported that a new chitosan derivative ZWC had excellent biocompatibility, comparing favorably with the precursor chitosan (LMCS) in hemocompatibility and with chitosan glutamate in the IP tissue responses [22]. Biological activity of chitosan is often attributed to the positive charges carried by the amine groups, which can electrostatically interact with cell membranes or circulating plasma proteins and lead to platelet adhesion/activation and thrombus formation [2], [24], [25]. Due to the ability to interact with serum proteins, chitosans activate macrophages and induce cytokine production [2]. Chitosan derivatives with reduced positive charge densities cause much lower platelet adhesion and aggregation than original chitosan [46]. Aqueous solubility of chitosan in physiological pH is also expected to play a role in biological responses, because chitosan precipitates can be subjected to phagocytic uptake and further stimulate macrophages. Therefore, we hypothesized that the good hemocompatibility of ZWC and the lack of pro-inflammatory effect might be related to the reduced amine contents of ZWC and/or the aqueous solubility at neutral pH.

To test this, we have first extended the evaluation of IP biocompatibility to a greater variety of chitosans, which include ZWC, glycol chitosan, and chitosan glutamate. ZWC and glycol chitosan are water-soluble in physiological pH, whereas chitosan glutamate precipitates at pH >6.5. Glycol chitosan and chitosan glutamate are comparable in the degree of deacetylation (i.e., amine content), whereas ZWC has less amines due to partial amidation with acid anhydride. The peritoneal cavity was used as a location to test biocompatibility of these chitosans, because of its well-known sensitivity to foreign insults, which results from the peritoneal defense mechanisms [47], [48]. We observed the most inflammatory responses associated with chitosan glutamate, although they were not as severe as in our previous experience with a rabbit model [27]. The peritoneal fluid cytology of this group showed eosinophilic chitosan debris surrounded by macrophages. These results were consistent with a previous study, where many macrophages with hyperplasia were observed in the mesenterium after IP injection of chitosan [33]. The animals treated with the water-soluble ZWC and glycol chitosan showed relatively low incidence of tissue lesions. Considering the difference in aqueous solubility of these chitosans, the inflammatory response caused by chitosan glutamate is likely to be primarily due to its low aqueous solubility in neutral pH, which results in precipitation of chitosan in the peritoneal cavity. Macrophages react directly to the precipitated materials and elicit inflammatory reactions, ultimately helping in degradation of the materials [49].

Chitosans have been shown to induce production of pro-inflammatory cytokines or chemokines from macrophages [27], [29], [31], [33], [50]. To examine if ZWC and glycol chitosan were intrinsically less bioactive than other chitosans, we then monitored the secretion of IL-1β, IL-6, TNF-α, and MIP-2 (murine functional homologue of IL-8 [51]) from peritoneal macrophages after treating with different chitosans. These cytokines or chemokines are responsible for both local and systemic inflammatory responses [49] and have been used in evaluating the safety of other chitosan based formulations [27], [30], [31], [49], [52]. Production of MIP-2, IL-6, and TNF-α in naïve macrophages was increased by treatment with chitosan glutamate but not with glycol chitosan, ZWC, or LMCS (Fig. 6A). Chitosan glutamate is not particularly more cytotoxic than others; therefore, the difference is unlikely due to the chemotactic effect of dead cells. A potential explanation is the high molecular weight of chitosan glutamate. Previous studies show that a relatively high molecular weight chitosan induces higher cytokine release from human keratinocytes [53]. Similarly, the relatively high molecular weight of chitosan glutamate (200 kDa), as compared to glycol chitosan (82 kDa), ZWC (15 kDa), and LMCS (15 kDa), may account for the relatively high pro-inflammatory effect of chitosan glutamate both *in vivo* and *in vitro*. The effect of the primary amine content on the intrinsic pro-inflammatory potential of chitosan is not readily apparent from the MAP panel assay given the lack of difference between ZWC and LMCS (Fig. 6A). ELISA detects a correlation between MIP-2 production and the amine content (LMCS>ZWC (An/Am = 0.3)>ZWC (An/Am = 0.7)), but the levels of MIP-2 are close to the basal level in all cases (Fig. 7A). According to these results, ZWC and glycol chitosan have relatively low potential to cause inflammatory reactions in the peritoneal cavity by themselves, and this property can be explained by their aqueous solubility and relatively low molecular weights.

Additionally, we consider another scenario of parenteral application, where chitosan is administered to tissues with lesions that attract activated macrophages. Interestingly, only ZWC suppressed the cytokine production from LPS-challenged macrophages significantly (Fig. 6B, Fig. 7B). Timed application of ZWC revealed that MIP-2 production stopped as soon as ZWC was applied (Fig. 8). There are at least two potential mechanisms by which ZWC counteracts the LPS stimulation. First, ZWC may bind the cell surface receptors and modify signaling pathways that regulate cytokine production. Second, ZWC may tightly bind to LPS and inactivate it. The fact that LPS pre-incubated with ZWC lost the ability to induce MIP-2 (Fig. 9) supports the second possibility. Moreover, ZWCs show much higher endotoxin content than other chitosans, further supporting that ZWC has high affinity to LPS. The mechanism of ZWC-LPS interaction is not yet clear, but the ZWC–mediated inactivation of LPS appears to be potent and irreversible, given that ZWC with such high endotoxin content did not activate naïve macrophages or induce inflammatory responses *in vivo*. MIP-2 production from the LPS-challenged macrophages decreased in the order of LMCS, ZWC (An/Am = 0.3), and ZWC (An/Am = 0.7) (Fig. 7B), indicating that this ability may be related to the amine content (inversely proportional to the amidation degree, An/Am ratio) in chitosan.

While we find that the interaction of LPS with ZWC is responsible for the reduced MIP-2 production from the LPS-challenged macrophages, we do not rule out the possibility of ZWC directly affecting the macrophages. Previous studies have shown at the molecular level that chitosan oligosaccharides modify the signaling mechanisms regulated by protein kinases and inhibit LPS-induced cytokine production [38]. A similar effect was observed with water-soluble chitosan or chitosan oligomers and LPS-activated RAW macrophages [39], [41].

The ability of ZWC to interact with LPS may be useful for a variety of biomedical applications. Coming from the cell wall of gram-negative bacteria, endotoxins are extremely potent stimulators of mammalian immune system. Endotoxins are a common cause of toxic reactions, and the level in parenteral products and water is strictly limited by the regulatory authorities [54]. As a scavenger of an endotoxin, ZWC may provide an efficient and cost-effective way of removing endotoxicin from pharmaceutical products.

In summary, ZWC showed excellent biocompatibility upon IP administration and had no pro-inflammatory effect on naïve macrophages. According to comparison with other chitosans, these properties may be attributable to the aqueous solubility at neutral pH and relatively low molecular weight of ZWC. Moreover, ZWC had unique ability to suppress cytokine production in LPS-challenged macrophages. The results suggest that ZWC has a strong affinity to LPS and inactivates its pro-inflammatory function. ZWC is a promising pharmaceutical excipient for parenteral use and may be also useful as an endotoxin scavenger.

## Supporting Information

Table S1 Chitosans used in this study and relevant properties.
(DOCX)
