# Supplementary material for: Zwitterionic Chitosan Derivative, a New Biocompatible Pharmaceutical Excipient, Prevents Endotoxin-Mediated Cytokine Release
Source: PLoS One. 2012 Jan 24;7(1):e30899. doi: 10.1371/journal.pone.0030899 (PMC3265529; doi:10.1371/journal.pone.0030899)
Supplement: Table S1 — Chitosans used in this study and relevant properties. (DOCX) [file pone.0030899.s001.docx]

**Zwitterionic Chitosan Derivative, a New Biocompatible Pharmaceutical Excipient, Prevents Endotoxin-mediated Cytokine Release.**

Gaurav Bajaj^1^, William G Van Alstine^2^, Yoon Yeo^1,3, *^

**Table S1.** Chitosans used in this study and relevant properties

|  | Description | Molecular weight | Degree of deacetylation (primary amine content) | Aqueous solubility at pH 7.4 |
| --- | --- | --- | --- | --- |
| Chitosan glutamate | Glutamate salt form | 200 kDa^a^ | 75–90% | Insoluble |
| Glycol chitosan | 2-hydroxyethylether derivative of chitosan | 82 kDa^a^ | 83% | Soluble |
| LMCS | Parent of ZWC | 15 kDa^a^ | 87% | Insoluble |
| ZWC (An/Am=0.3) | <29%^b^ | ~15 kDa | >58% | Soluble |
| ZWC (An/Am=0.7) | >52%^b^ | ~15 kDa | <35% | Soluble |

1. Provided by the manufacturer.
2. Estimated from the previous study [[22](#_ENREF_22)].
